# Assessing the Impact of Various Decontamination Instruments on Titanium and Zirconia Dental Implants: An In Vitro Study

**DOI:** 10.3390/dj12050136

**Published:** 2024-05-09

**Authors:** Louisa Vierling, Chun Ching Liu, Daniel Wiedemeier, Andrea Gubler, Patrick R. Schmidlin

**Affiliations:** 1 Clinic of Conservative and Preventive Dentistry, Division of Periodontology and Peri-Implant Diseases, Center of Dental Medicine, University of Zurich, 8032 Zurich, Switzerland; louisa.vierling@uzh.ch (L.V.); chunching.liu@zzm.uzh.ch (C.C.L.); andrea.gubler@zzm.uzh.ch (A.G.); 2 Statistics Group, Center of Dental Medicine, University of Zurich, 8032 Zurich, Switzerland; daniel.wiedemeier@zzm.uzh.ch

**Keywords:** dental implants, particles, surface properties, abrasion, titanium, zirconium

## Abstract

This study investigates the impact of various instrumentation techniques on material removal and surface changes in titanium (Ti)- and zirconia (Zr) implant discs. Ti- and Zr discs were subjected to standardized experiments using various instruments including airflow, ultrasound, carbide, and diamond burs. Instrumentation was performed for 60 s with continuous automatic motion. Abrasion and changes in surface roughness were assessed using profilometry, while scanning electron microscopy was used to examine morphological changes and particle size. Carbide burs predominantly caused abrasion on Ti discs, while diamond burs caused more abrasion on Zr discs. The Ti discs were more susceptible to surface changes. However, among the materials tested, machined Zr discs treated with diamond burs produced the largest particle. In certain cases, a statistical significance (*p* < 0.05) was observed between the groups, while in others, there was no considerable difference among the means (*p* > 0.05). These results highlighted the statistical significance of our findings. These results found diverse alterations in surface characteristics of Ti- and Zr discs due to different instruments, with carbide and diamond burs causing notable effects. The findings highlight the need for a careful balance between promoting healing and minimizing harm during implantoplasty.

## 1. Introduction

In modern dentistry, dental implants are widely used, offering patients a solution for tooth replacement. Titanium (Ti) and zirconia (Zr) are two commonly used implant materials, with zirconia rapidly gaining popularity owing to their excellent aesthetic, mechanical, and biological properties [1]. However, maintaining peri-implant health remains crucial for ensuring the long-term success of dental implants. Peri-implantitis has emerged as a common biologic complication in implantology, effecting 18.5% at the patient level and 12.8% at the implant level, specifically titanium implants [2]. Among the diverse array of decontamination instruments and methods available, understanding their respective effects on different implant materials is crucial for optimizing treatment outcomes. 

Implantoplasty has emerged as a promising technique for addressing per-implantitis, involving surgical intervention for adequate access to clean, polish and mechanically modify the infected implant surface to eliminate the biofilm while preserving implant integrity [3,4,5]. This rather aggressive method entails mechanical elimination of the textured implant surface by using different instruments like diamond or carbide burs [6,7]. It results in reduced surface roughness and subsequent plaque adhesion to the implant [8] by removing the most retentive macro- and micro-morphological aspects of the outer surface [9], with its long-term goal to facilitate the healing process, resolve inflammation and prevent further bone loss [6]. While implantoplasty is a well-established technique, its relevance persists, particularly with the growing popularity of zirconia implants.

Particle release during implantoplasty has raised concerns regarding its cytotoxic and genotoxic effects [3]. It can induce inflammation and further the progression of peri-implantitis [10,11]. Notably, studies have shown higher concentrations of Ti-particles in the tissues surrounding Ti-implants in patients with peri-implantitis compared to controls with periodontitis [11]. These particles can be detected at varying distances from the implant, ranging from 0.4 to 4 mm [12], and may even disseminate to more distant parts of the body [13]. Additionally, metallic debris in affected tissues can compromise the aesthetic appearance of the peri-implant mucosa [14] or trigger hypersensitivity reactions [15].

Surface characteristics of the implant, including surface roughness, play a crucial role in osseointegration and bacterial adhesion, thus necessitating careful consideration when selecting decontamination instruments. While increasing the surface area of an implant through amplified surface roughness can facilitate the healing process [16], rougher surfaces may also promote bacterial colonization and epithelial cell attachment once exposed. This results in the formation of biofilm and calculus, making it more challenging to prevent and control peri-implantitis as a pathologic sequela [17,18].

In this context, the objective of this study was to assess the effects of commonly used instruments on the surface characteristics of Ti and Zr implants. By evaluating surface abrasion and surface roughness alterations, we aim to provide valuable insights into the selection of dental implant materials and cleaning techniques on these two implant materials. The null hypothesis assumed that different instruments would induce similar abrasion, changes in surface roughness, and material loss, regardless of the implant material and surface type.

## 2. Materials and Methods

### 2.1. Ti and Zr Discs 

In this study, Ti (Straumann, Basel, Switzerland) and Zr discs (Straumann, Basel, Switzerland) were used, each featuring two distinct surface types: acid-etched and machined. While titanium discs used in this study are composed of titanium grade IV, zirconia discs entirely consist of yttria-stabilized zirconia. The acid etching of the discs was carried out by Straumann prior to testing, using a large grit sand-blasting process with corundum particles. This was followed by an acid-etching bath with a mixture of HCI/H_2_SO_4_ for several minutes.

The discs exhibited varying dimensions; acid-etched titanium and machined zirconia had a diameter of 15 mm and a thickness of 0.7 mm, while titanium machined, and zirconia acid-etched discs featured a 5 mm diameter with the same 0.7 mm thickness. 

To secure the Ti acid-etched and Zr discs with a machined surface, they were affixed to standard scanning electron microscopy (SEM) specimen mounts (diameter: 12.7 mm, slotted head, and 3.1 mm tapered end pin) using Loctite 480 black glue (Henkel, Dusseldorf, Germany). The Ti-discs with machined surfaces and Zr-discs with acid-etched surfaces were embedded in the SEM specimen mounts through a milling process, with a 5.0 mm diameter and 0.9 mm depth, and bonded using the same glue. The discs were categorized into 16 groups based on the implant material, surface finish, size and the instrument used (Table 1).

### 2.2. Sample Numbers 

A total of 64 titanium and 64 zirconia discs were used, with each material group consisting of 32 acid-etched and 32 machined surfaces. This resulted in a total of 128 discs, with 64 from each surface condition across both materials. Each condition was subjected to evaluation under four distinct treatments. Consequently, this experimental design resulted in eight samples per instrument/material/surface condition combination.

### 2.3. Instrumentation Protocols

Four different instruments were used, including diamond (379.314.014 VPE) and carbide burs (H379.204.014 VPE 5) (Komet Dental, Lemgo, Germany), an ultrasonic scaling device (EMS PIEZON handpiece, Airflow Prophylaxis Master; EMS Dental, Nyon, Switzerland) with a steel tip (EMS PIEZON Perio Slim Tip), or an airflow device (Airflow Prophylaxis Master) used in conjunction with 25 µm particle-size glycine airflow powder (Airflow Powder Perio) from EMS Dental (Nyon, Switzerland). 

For the treatment with diamond and carbide burs, the handgrip of the contra-angle handpiece (Model 150 ISM, MicroMega, Besancon, France) with the respective bur was secured in an adjustable device, allowing for controlled vertical and horizontal movements. To achieve this, the SEM specimen mounts with glued discs were positioned on the flat surface of a portable table with changeable X- and Y-axes. The tip of the bur made tangential contact at a 40° angle with the disc surface. This setup ensured consistent and standardized instrumentation. Prior to each treatment, the handpiece was moved using a motor and set to a treatment distance of 10 mm by applying a constant force of 100 g, calibrated with a spring (Mettler Delta Range PC 440, Mettler-Toledo, Greifensee, Switzerland). Each bur was used for 60 s, operated at a speed of 40,000 rpm with water irrigation, treating a maximum of 8 discs within its assigned group. In total, 8 burs were used, with each bur dedicated exclusively to one specific group. This comprised 4 carbide and 4 diamond burs, ensuring consistency and accuracy with each group. Similarly, an ultrasonic scaler was utilized for 60 s on each disc, using continuous movements and 100% water irrigation, operating at a power of 4.8 W. The operator selected the angulation of the steel tip, while the SEM specimen mounts were held straight by a SEM pin-stub holder during the treatment. The same ultrasonic scaler and steel tip was used for all discs. The airflow device was employed for 60 s on each disc, maintaining a constant distance of 4 mm. Following the manufacturer’s recommendations, it was operated with a 100% water supply and at 50% power. The device was secured via a custom-made clamp connected to the SEM pinstub holder, set at a 40° angle to the disc surface. The SEM specimen mounts were held straight using a SEM pinstub holder. 

The instrumentation with all four instruments was performed by the same operator. The operator underwent calibration before the study. This involved standardized training to ensure the consistent and proficient use of all four instruments, including instructions on proper handling, pressure application and movement patterns. Before performing the main study, previous experiments were conducted to verify the integrity.

### 2.4. Profilometric Analysis and SEM Analysis

To assess changes in surface roughness, discs were analyzed using a contact profilometer (Taylor Hobson, AMETEK GmbH, Weiterstadt, Germany). Six measurements were taken per disc before and after treatment, covering a preset analysis length of 1.5 × 1.5 mm^2^ in 0.5 mm increments along the Y-axis. As the discs displayed continuous parallel grooves after the instrumentation, three measurements were conducted in the direction of instrumentation, with the remaining three taken at a 90° angle to the instrumentation direction. Surface abrasion of each disc was measured using a perthometer (Mahr S2; Perthometer, Thalwil, Switzerland). A total of five measurements, spanning a length of 1.5 mm with 250 μm increments along the X-axis, were analyzed. To evaluate surface changes resulting from instrumentation, the surfaces of two discs per group were examined using SEM (GeminiSEM450, Carl Zeiss, Oberkochen, Germany). Before analysis, the samples were coated with a 6 nm layer of gold. Magnifications of 100×, 1000×, 5000×, 10,000×, and 10 kV were utilized. To determine the size and shape of the released particles, three discs per group were examined. An adhesive tape was used to collect particles from the disc surfaces, and these particles were subsequently analyzed using SEM at the same magnification level.

### 2.5. Statistical Analysis 

Descriptive statistics (mean ± standard deviation) were calculated and, accordingly, reported. Moreover, the groups (Table 1) were compared in terms of surface abrasion and change in surface roughness using Kruskal–Wallis omnibus tests, followed by Conover’s post hoc tests. To account for multiple testing, the resulting *p*-values were adjusted according to Benjamini and Yekutieli [19]. A *p*-value of less than 0.05 was considered to indicate statistical significance. All statistical analyses and plots were computed using the statistical software R version 4.2.3 [20], including the packages tidyverse [21], PMCMR [22], and PMCMRplus [23].

## 3. Results

### 3.1. Surface Abrasion

**Figure 1:**
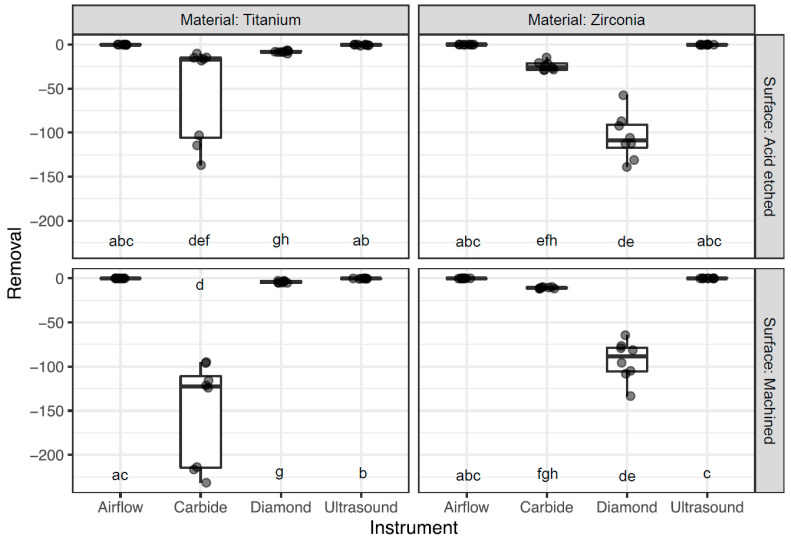
Boxplots displaying the average removal per material, surface, and instrument. Groups with at least one common letter are not significantly different from each other (*p* > 0.05), considering both vertical and horizontal comparisons. The comparisons between instruments were made across all four material–surface windows in order to show every possible comparison of the sixteen groups.

Figure 1 and Table 2 detail the amounts of material removed from the discs following the use of different instruments. 

The greatest amount of material removal was observed on machined Ti discs treated (TM) with carbide burs, resulting in an average removal of −151.8 ± 58.3 µm. Carbide burs also led to material removal on Ti acid-etched discs (TA), with an average of −53.6 ± 54.3 µm. However, the same burs resulted in less material removal from Zr discs. Notably, the acid-etched Zr discs (ZA) exhibited an abrasion of −24.4 ± 5.04 µm, while the machined Zr discs (ZM) displayed a reduction of −10.8 ± 0.8 µm. Conversely, the use of diamond burs resulted in substantial material removal from the Zr discs. Both ZA and ZM discs exhibited a removal of −104.7 ± 25.9 and −92.9 ± 22.1 µm, respectively, after treatment. In contrast, ultrasound and airflow had minimal effects on the material removal for all disc types.

### 3.2. Change in Surface Roughness

**Figure 2:**
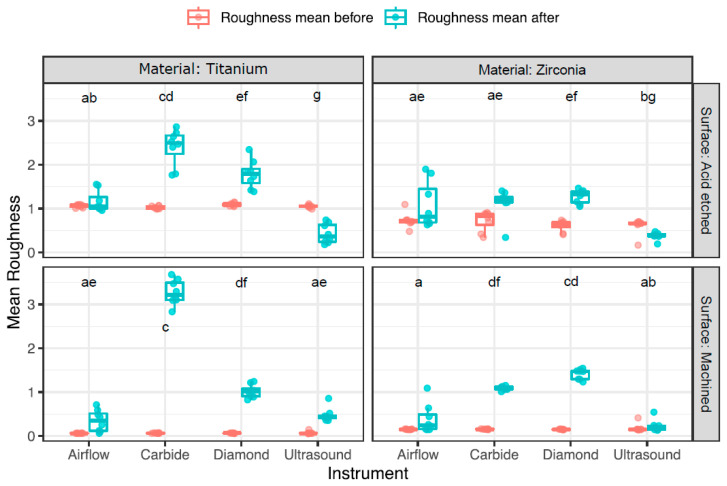
Boxplots displaying the average surface roughness before and after treatment per material, surface, and instrument. Groups with at least one common letter are not significantly different from each other (*p* > 0.05), considering both vertical and horizontal comparisons. The comparisons between instruments were made across all four material–surface windows in order to show every possible comparison of the sixteen groups.

The results of the profilometrically assessed surface roughness before and after treatment are depicted in Figure 2 and Table 3. Initially, the untreated discs exhibited variable surface roughness, ranging from rather smooth surfaces on machined discs, measuring 0.1 ± 0.01 µm on titanium and 0.2 ± 0.03 µm on zirconia discs, to moderately rougher surfaces on acid-etched discs, with zirconia measuring 0.7 ± 0.2 µm and titanium discs measuring 1.1 ± 0.04 µm.

After treatment, the discs exhibited a range of surface roughness results, depending on the surface and treatment modality. TM discs treated with carbide burs displayed the highest surface roughness, with a mean difference of 3.2 ± 0.3 µm. Additionally, they caused an elevated surface roughness of 2.4 ± 0.4 µm on TA discs. The diamond burs increased the surface roughness, particularly on the ZM discs, leading to an increase of 1.3 ± 0.1 µm. In comparison, the airflow had a minimal effect on the surface roughness changes, and no significant difference among the four materials was observed. However, this led to the most notable increase in the ZA discs. In contrast, ultrasound treatment rather smoothed the surfaces of acid-etched Ti- and Zr-discs, reducing the overall surface roughness by 0.6 ± 0.2 and 0.2 ± 0.1 µm, respectively. 

### 3.3. Scanning Electron Microscopy (SEM) Particle Analysis 

**Figure 3:**
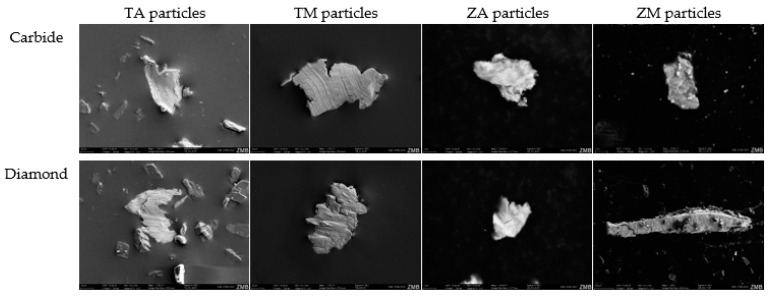
SEM images of disc particles released using carbide and diamond burs.

The evaluation of the particles from Ti and Zr discs subjected to airflow or ultrasound did not yield any exploitable results, rendering the analysis unsuccessful. Therefore, the analysis was limited to particles regularly found on the discs treated with carbide or diamond burs (Figure 3).

This study revealed the presence of acid-etched Ti-particles treated with carbide burs of sizes up to 105.8 µm. Smaller particles, measuring up to 88.2 µm, were detected when treated with diamond burs. On TM discs, the largest detectable particles measured 152.9 µm when treated with carbide burs, and 123.5 µm when treated with diamond burs. The particles obtained from the Zr-discs were slightly smaller. When treated with carbide burs, ZA discs contained particles up to 5.3 µm in size, while those treated with diamond burs had particles up to 4.1 µm. However, ZM discs exhibited the largest particles of all, measuring 800 µm when treated with diamond burs. Conversely, the particles treated with carbide burs were significantly smaller, measuring 8.9 µm in length.

### 3.4. SEM Analysis of Disc Surface 

**Figure 4:**
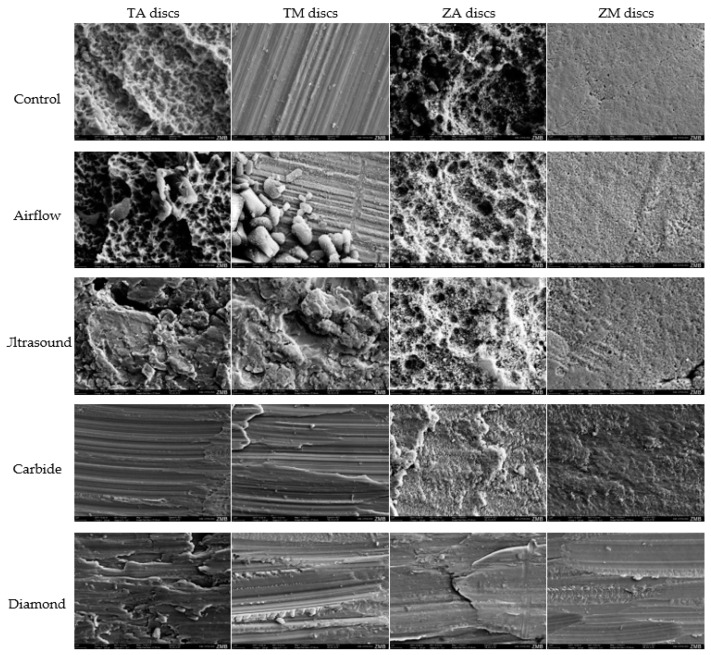
SEM images of disc surfaces after treatment per material, surface, and instrument.

Optical analysis of the changes in surface topography was conducted using SEM (Figure 4). Overall, airflow had a minimal impact on disc morphology. However, traces of glycine powder were observed, particularly on machined Ti discs. Ultrasound treatment led to visible surface roughening of the machined Ti discs, resulting in loss of the characteristic surface morphology. The use of carbide burs caused surface alterations of the Ti discs; however, the effects of the treatment varied depending on the direction in which the burs were applied. When used in one direction, the surface was smooth, whereas perpendicular application resulted in the formation of elevations. The diamond burs induced significant changes, roughening the surface, and creating an irregular surface, especially on the Ti-discs. Initially, the machined Zr discs had a relatively even surface, but treatment, particularly with diamond burs, resulted in optical irregularities on the surface.

## 4. Discussion

In this study, we conducted a comprehensive comparative examination of the effects of ultrasound, airflow, diamond, and carbide burs on the surface characteristics of Ti and Zr discs. These instruments are already known to be capable of modifying the surface roughness and causing abrasion, potentially leading to the release of implant material particles. Therefore, this study specifically evaluated the changes induced by these instruments on discs with different roughnesses and material properties.

The study’s findings reject the null hypothesis that different instruments would cause similar changes in surface roughness and abrasion, and that there would be no significant difference between the groups. The outcomes obtained from multiple assessment techniques, including profilometry and SEM analysis, demonstrated that the use of all instruments led to varying degrees of reduction in loss of the typical surface characteristics in all discs. This evidence indicates that the choice of instrument utilized for implant treatment can significantly impact the surface roughness of both Ti and Zr implant materials. Moreover, both the type of implant material and instruments used for treatment influenced the changes in surface roughness and particle release.

Profilometric analysis revealed that the carbide and diamond burs caused considerably more surface abrasion on both Ti and Zr implants than on the ultrasonic scaler and airflow device. While diamond burs had a greater effect on Zr discs, carbide burs induced more surface changes in Ti discs. Among all the instruments tested, carbide burs, particularly on the Ti discs, resulted in the roughest surface. 

Notably, changes in the surface roughness can impact the success of implant integration by affecting cell adhesion and proliferation on the implant surface. An animal study has demonstrated that decreased surface roughness may result in reduced bone healing attributed to decreased wettability and biocompatibility [24,25,26]. Conversely, smoother surfaces tend to hinder biofilm adhesion [8], while rougher surfaces can facilitate it [17]. This is supported by a former in vivo study that proposed a threshold surface roughness (Ra = 0.2 µm), below which no additional reduction in bacterial accumulation is anticipated [27]. However, an elevation beyond this threshold concurrently led to an increase in plaque accumulation, consequently increasing the risk of peri-implantitis [28]. In our study, irrespective of the instrument or material used, all discs presented a surface roughness exceeding 0.2 µm after instrumentation. This implies that every disc presents an elevated risk of plaque formation. 

Furthermore, studies have indicated that plaque accumulation on Zr implants seemed lower as compared to Ti implants, which was assumed to potentially decrease biological complications such as peri-implantitis [29,30]. Moreover, Zr can undergo a phase transformation, shifting from a tetragonal to a monoclinic phase under stress [31]. This leads to an expansion in grain volume at the crack tip, counteracting crack growth [32,33]. Conversely, in a humid environment such as the oral cavity, this transformation can contribute to increased surface roughness and micro-cracks, which makes it more prone to bacterial adhesion [33].

In our study, the type of implant material had a critical influence on the quantity and size of the released particles. The Ti implants released substantially more and larger particles than the Zr discs, except for the ZM discs treated with diamond burs. This finding is consistent with a previous study suggesting that Zr implants release fewer particles than Ti-implants [34,35]. But the implantoplasty-induced release of implant particles may cause a reduction in both diameter and thickness of the implant, leading to a diminished fracture resistance [36]. These findings have important clinical implications, particularly when considering the selection of implant materials. However, a study performed by Martins et al. showed that implantoplasty in combination with osteoplasty and an apically positioned flap is a safe treatment option for peri-implantitis [5], with low rates of peri-implantitis recurrence and implant loss [37].

But also, the choice of the instrument can contribute to an increased release of particles from the implant surface. This is evident from the identification of the largest detectable particles on the ZM discs treated with carbide burs. Moreover, carbide burs were responsible for releasing the largest particles among all the tested discs. In contrast, discs treated with airflow and ultrasound instruments exhibited no detectable particles, leading to the conclusion that either no particles were released, or that they were too small to be identified and evaluated within the limitations of our methodological setup. However, different studies have shown that particles from Ti implants can be released due to treatment with an ultrasound handpiece, with sizes ranging between 6 and 8 µm [10,38]. To date, no studies on ultrasound-induced particle release from Zr-implants have been identified by the authors to support the results herein; in addition, there is no previous research exploring particle release using airflow devices.

The biological significance of our findings lies in the fact that multiple studies have demonstrated that detached Ti implant particles can penetrate the oral epithelial barrier and cause bacterial accumulation around the implant [39,40]. Moreover, Ti particles have been associated with peri-implant inflammation [41], osteolysis [42], peri-implantitis, and ultimately, implant loosening [43,44]. Conversely, Zr particles have been found to induce less inflammation and bone resorption than Ti particles, indicating a claimed better biocompatibility [45]. It seems advisable to use less or noninvasive cleaning and/or polishing techniques to at least minimize particle release, particularly to avoid the use of carbide and diamond burs, if not absolutely necessary.

Finally, it remains important to critically acknowledge the limitations of this in vitro study when interpreting its biological implications for clinical practice. First, the use of flat-surfaced discs instead of regular implants may have influenced the results, as the cylindrical shape and threads of the implants could additionally impact surface flattening and abrasion. Additionally, the presence of a biofilm on the implant surface can influence the extent of abrasion or surface changes. Furthermore, REM analysis was only conducted on three discs per group, providing a rough approximation based on a standardized protocol. This analysis does not accurately represent the actual particle release of the disc materials and only investigates the immediate effects of surface treatment. Therefore, future studies should investigate the long-term impact of implant success rates and clinical outcomes in the context of implantoplasty.

## 5. Conclusions

This in vitro study provides valuable insights into the distinct effects of various instrumentation methods on Ti- and Zr implant materials. It highlights the important role of instrument selection during implantoplasty. It is crucial to recognize that the tested materials do not respond similarly to the same procedures, with Ti-implants being more susceptible to surface modification than Zr implants. Also, the choice of instrument had an impact, showing that carbide and diamond burs pronounced abrasion, particularly on Ti discs. These alterations in surface morphology hold important implications for implant success, emphasizing the necessity of thoughtful instrument choice in clinical settings. Furthermore, our findings emphasize the importance of reducing particle release during implantoplasty, especially by avoiding rather aggressive burs, to reduce the risk of complications. Our findings underscore the delicate balance between promoting healing and avoiding potential harm when different implant materials are used during implantoplasty.

## Figures and Tables

**Table 1:** Groups based on material, surface, instrumentation, and measurements.

64 Ti Discs, 64 Zr Discs (128 Discs) (per Group n = 8)	Ti Discs		Zr Discs	
	Acid-Etched Surface (TA)	Machined Surface (TM)	Acid-Etched Surface (ZA)	Machined Surface (ZM)
Carbide burs	Diameter = 15 mm Thickness = 0.7 mm	Diameter = 5 mm Thickness = 0.7 mm		Diameter = 15 mm Thickness = 1.4 mm
Diamond burs				
Ultrasonic scaler				
Airflow with glycine powder				

**Table 2:** Removal of disc material after instrumentation.

Disc	Instrument	Mean ± Sd (µm)	Median ± IQR (µm)
TA	Airflow	−0.2 ± 0.2	−0.1 ± 0.2
TA	Carbide	−53.6 ± 54.3	−17.4 ± 90.9
TA	Diamond	−8.2 ± 1.2	−8.4 ± 1.2
TA	Ultrasound	−0.5 ± 0.5	−0.3 ± 0.8
TM	Airflow	−0.1 ± 0.1	0 ± 0.1
TM	Carbide	−151.8 ± 58.3	−122.7 ± 103.7
TM	Diamond	−4.1 ± 1.1	−4.4 ± 1.9
TM	Ultrasound	−0.3 ± 0.2	−0.2 ± 0.02
ZA	Airflow	−0.1 ± 0.2	0 ± 0.3
ZA	Carbide	−24.4 ± 5.04	−25.6 ± 7.1
ZA	Diamond	−104.7 ± 25.9	−108.9 ± 26.2
ZA	Ultrasound	−0.2 ± 0.2	−0.2 ± 0.4
ZM	Airflow	−0.1 ± 0.1	−0.1 ± 0.2
ZM	Carbide	−10.8 ± 0.8	−10.9 ± 1.3
ZM	Diamond	−92.9 ± 22.1	−88.5 ± 27.2
ZM	Ultrasound	−0.03 ± 0.1	0 ± 0.03

**Table 3:** Surface roughness parameters before and after instrumentation and the difference between them.

Disc	Instrument	Mean ± Sd (µm)			Median ± IQR (µm)		
		Before	After	Diff.	Before	After	Diff.
TA	Airflow	1.1 ± 0.03	1.2 ± 0.2	0.1 ± 0.2	1.1 ± 0.5	1.1 ± 0.3	−0.03 ± 0.2
TA	Carbide	1 ± 0.03	2.4 ± 0.4	1.4 ± 0.4	1 ± 0.6	2.5 ± 0.4	1.5 ± 0.5
TA	Diamond	1.1 ± 0.04	1.8 ± 0.3	0.7 ± 0.3	1.1 ± 0.5	1.8 ± 0.3	0.7 ± 0.4
TA	Ultrasound	1.1 ± 0.04	0.4 ± 0.2	−0.6 ± 0.2	1.1 ± 0.02	0.4 ± 0.4	−0.7 ± 0.4
TM	Airflow	0.1 ± 0.004	0.4 ± 0.3	0.3 ± 0.2	0.1 ± 0.004	0.3 ± 0.4	0.3 ± 0.4
TM	Carbide	0.1 ± 0.01	3.2 ± 0.3	3.2 ± 0.3	0.1 ± 0.01	3.2 ± 0.4	3.1 ± 0.4
TM	Diamond	0.1 ± 0.004	1 ± 0.2	1 ± 0.2	0.1 ± 0.003	1 ± 0.2	1 ± 0.2
TM	Ultrasound	0.1 ± 0.03	0.5 ± 0.2	0.4 ± 0.2	0.1 ± 0.01	0.4 ± 0.1	0.4 ± 0.1
ZA	Airflow	0.7 ± 0.2	1.1 ± 0.5	0.4 ± 0.4	0.7 ± 0.1	0.8 ± 0.8	0.2 ± 0.7
ZA	Carbide	0.7 ± 0.2	1.1 ± 0.3	0.4 ± 0.2	0.8 ± 0.3	1.2 ± 0.1	0.4 ± 0.2
ZA	Diamond	0.6 ± 0.1	1.3 ± 0.2	0.7 ± 0.2	0.6 ± 0.1	1.3 ± 0.3	0.7 ± 0.3
ZA	Ultrasound	0.6 ± 0.2	0.4 ± 0.1	−0.2 ± 0.1	0.6 ± 0.3	0.4 ± 0.04	−0.3 ± 0.1
ZM	Airflow	0.2 ± 0.01	0.4 ± 0.3	0.2 ± 0.3	0.2 ± 0.01	0.2 ± 0.3	0.1 ± 0.3
ZM	Carbide	0.2 ± 0.01	1.1 ± 0.1	1 ± 0.05	0.2 ± 0.01	1.1 ± 0.1	0.9 ± 0.1
ZM	Diamond	0.2 ± 0.01	1.4 ± 0.1	1.3 ± 0.1	0.2 ± 0.01	1.5 ± 0.2	1.3 ± 0.2
ZM	Ultrasound	0.2 ± 0.1	0.2 ± 0.1	0.04 ± 0.2	0.2 ± 0.01	0.2 ± 0.1	0.03 ± 0.1

## Data Availability

The raw data supporting the conclusions of this article will be made available by the authors on request.
